# Public Pharmacists' Association

**Published:** 1920-04-24

**Authors:** 


### Public Pharmacists' Association.

### A PLEA FOR FURTHER LECTURES.

A meeting of the Association was lately held at St. Bride's Institute, Ludgate Circus, London. Mr. J. L. P. Hollingworth, F.L.S. (Victoria Hospital, Chelsea), presided over a representative gathering. Mr. A. E. Mills, M.P.S., Royal United Hospital, Bath, was elected a member of the Association.

The evening was devoted to a lecture by Dr. Stanley Wyard, M.D., M.R.C.P., Pathologist to the Children's Hospital, Chelsea, and the Bolingbroke Hospital, etc., on "Pathological Specimens and their Preparation." The lecturer at the outset explained that he proposed to deal chiefly with the preparation of the various staining solutions, the manner in which these acted, and the preparation of the specimens. Dr. Wyard explained that there were three groups of stains in use—viz. (*a*) metallic (silver and gold); (*b*) vegetable (logwood) acid solution used and precipitated with alkali; (*c*) aniline dyes as crystal violet, gentian violet, eosin (water-soluble and alcohol-soluble); fuchsua (acid and basic), the former soluble in water, the latter not being soluble; methylene blue, etc. "Acid Fast" preparations are those from which the dye such as fuchsua is not removed by treatment with sulphuric acid, 25 per cent. "Gram Positive" specimens retain the stain of grammes iodine solution, whilst "Gram Negative" do not.

Dr. Wyard explained the use of mordants in connection with the subject, also colloids and colloidal precipitation and the action of asbestos therewith. Brilliant green and its use for the detection of typhoid bacilli was also described.

Mr. Hollingworth, in proposing a hearty vote of thanks to the lecturer, referred to the need for institutional pharmacists to keep themselves up to date in their knowledge of the newer methods in medicine and surgery so far as these affected their department.

Mr. T. S. Goodall (Skin Hospital), in supporting, expressed the hope that the Council of the Public Pharmacists' Association would arrange more of these useful and educational evening meetings, for which they had achieved a reputation.

Dr. Wyard replied to several questions, and concluded by expressing his pleasure at meeting members of the pharmaceutical profession.

